# *PAX5-KIAA1549L*: a novel fusion gene in a case of pediatric B-cell precursor acute lymphoblastic leukemia

**DOI:** 10.1186/s13039-015-0138-3

**Published:** 2015-07-08

**Authors:** Stefanie Anderl, Margit König, Andishe Attarbaschi, Sabine Strehl

**Affiliations:** CCRI, Children’s Cancer Research Institute, St. Anna Kinderkrebsforschung e.V., Vienna, Austria; Department of Pediatrics, St. Anna Children’s Hospital, Vienna, Austria; Medical University of Vienna, Vienna, Austria

**Keywords:** B-cell precursor acute lymphoblastic leukemia, *PAX5* fusion, *KIAA1549L*

## Abstract

**Background:**

In B-cell precursor acute lymphoblastic leukemia (BCP-ALL) PAX5, a transcription factor pivotal for B-cell commitment and maintenance, is frequently affected by genetic alterations. In 2-3 % of the cases *PAX5* rearrangements result in the expression of oncogenic fusion genes. The encoded chimeric proteins consist of the N-terminal PAX5 DNA-binding paired domain, which is fused to the C-terminal domains of a remarkable heterogeneous group of partner proteins.

**Results:**

Employing fluorescence in situ hybridization and molecular methods *PAX5-KIAA1549L* was identified as novel fusion gene in a case of pediatric BCP-ALL.

**Conclusion:**

Our report underlines the high diversity of *PAX5* fusion partners in BCP-ALL and we describe the second involvement of *KIAA1549L* in a genetic rearrangement in acute leukemia.

**Electronic supplementary material:**

The online version of this article (doi:10.1186/s13039-015-0138-3) contains supplementary material, which is available to authorized users.

## Background

Acute lymphoblastic leukemia (ALL) is the most common childhood malignancy and one of the leading causes of cancer-related death in children and young adults. ALL is characterized by a plethora of somatic mutations, which are involved in the pathogenesis and progression of the disease [1]. In this regard, in childhood B-cell precursor ALL (BCP-ALL) PAX5, a transcription factor crucial for B-cell commitment and maintenance [2], is frequently affected by genetic alterations comprising deletions, point mutations, amplifications, and chromosomal rearrangements [3–7]. The latter occur in 2-3 % of the cases and result in the expression of chimeric proteins consisting of the PAX5 DNA-binding paired domain (PD) at the N-terminus, fused to the C-terminal domains of a highly diverse group of partner proteins [3–5, 8]. A multitude of functionally different PAX5 partners, including transcription factors, chromatin regulators, protein kinases, and structural proteins, have been identified and to date more than 20 in-frame *PAX5* fusions have been described [3–5, 8–15]. The unifying feature of all PAX5 chimeras, which are generally suggested to act as aberrant transcription factors antagonizing wild-type PAX5 function, is the retention of the DNA-binding domain [2, 3, 9, 16–18]. However, the partner protein appears to modulate the function of the different PAX5 fusion proteins, suggesting differences in the development of the respective leukemia [19, 20]. In this work we describe a novel *PAX5* rearrangement in a case of pediatric BCP-ALL in which *PAX5* is fused to *KIAA1549L*.

## Case presentation

A 2.2-year old girl was diagnosed with BCP-ALL. The bone marrow and the peripheral blood showed an infiltration with 94 % and 89 % blast cells of L1 morphology, respectively. Immunophenotyping demonstrated CD19+, CD10+, cyCD22+, HLA-DR+, TdT+, cyIgM+, mIgM-, MPO-, CD34-, CD14-, CD13-, CD33-, cyCD3-, and CD7- blast cells with coexpression of the myeloid marker CDw65 on 20 % of them. Cytogenetic and molecular analyses showed a normal 46,XX[20] karyotype and negativity for the *ETV6-RUNX1*, *KMT2A-AFF1* (*alias MLL-AF4*), *BCR-ABL1*, and *TCF3-PBX1* fusion genes.

The patient was enrolled in the ALL-BFM 95 study (ClinicalTrials.gov Identifier: NCT00411541) and treated in the intermediate risk arm of the protocol [21, 22]. The patient was randomized into the methotrexate and cytarabine (MCA) protocol, which included an intensification of the extracompartement phase with an intermediate dose cytarabine in addition to high-dose methotrexate. For maintenance therapy the patient was randomized into the treatment arm, which received an intensified therapy with additional pulses of dexamethasone and vincristine. The patient is in long-term remission 8.6 years after initial diagnosis.

## Material and methods

### Cytogenetic and fluorescence in situ hybridization (FISH) analysis

Cytogenetic and FISH analyses were performed according to standard techniques. FISH was conducted using *PAX5* exon-specific cosmid probes cos-hPAX5-1 (exons 2–5) and cos-hPAX5-3 (exons 9-10) as previously described [4].

### Rapid amplification of cDNA ends (RACE) and reverse transcription-polymerase chain reaction (RT-PCR)

RNA isolation was performed with the Qiagen RNeasy Mini-Kit (Qiagen, Germany) following the manufacturer’s instructions. RACE ready cDNA was generated using 220 ng total RNA input and the SMARTer™ RACE cDNA Amplification Kit (Clontech/Takara, France). 3′-RACE-PCR and 3′-nested RACE-PCR were performed with the Advantage 2 PCR Kit (Clontech/Takara, France) and standard PCR reagents (Sigma, Austria), respectively.

RT-PCR for the detection of *PAX5*-*KIAA1549L* transcripts was performed according to standard procedures using primers located in exon 5 of *PAX5* (PAX5ex5_F1 5′-TACTCCATCAGCGGCATCC-3′) and exon 20 of *KIAA1549L* (KIAA1549Lex20_R2: 5′-AACACGTAGGCATGGGAAAC-3′). Amplification products were directly sequenced (Microsynth AG, Austria) and sequence analysis was conducted using the CLC Main Workbench 6.0 (CLC bio, Denmark).

### Reference sequences, exon nomenclature, and protein analysis

The exon nomenclature used corresponds to that of the NCBI database (accessed December 2014) and the reference sequences NM_016734.2 for *PAX5* and NM_012194 for *KIAA1549L* were used. Protein motif search was performed with Psort II Prediction [23] and NCBI_Protein [24]. The conservation of KIAA1549L across species was derived from NCBI_HomoloGene [25].

### Detection of copy number alterations (CNAs)

Genome-wide CNAs were determined using the CytoScan HD array platform (Affymetrix, USA). This array platform contains 2.67 million probes, including 1.9 million copy number probes and 0.75 million SNP probes. Array analysis and interpretation was conducted with the Chromosome Analysis Suite (ChAS 2.0.0) software (Affymetrix, USA). Genome segment filters were set to a marker count of 50 and a size of 50 kb for gains and losses. The ChAS Browser Annotations version NetAffx built 32.3 (UCSC genome assembly hg19) was used for analysis.

## Results and discussion

**Fig. 1:**
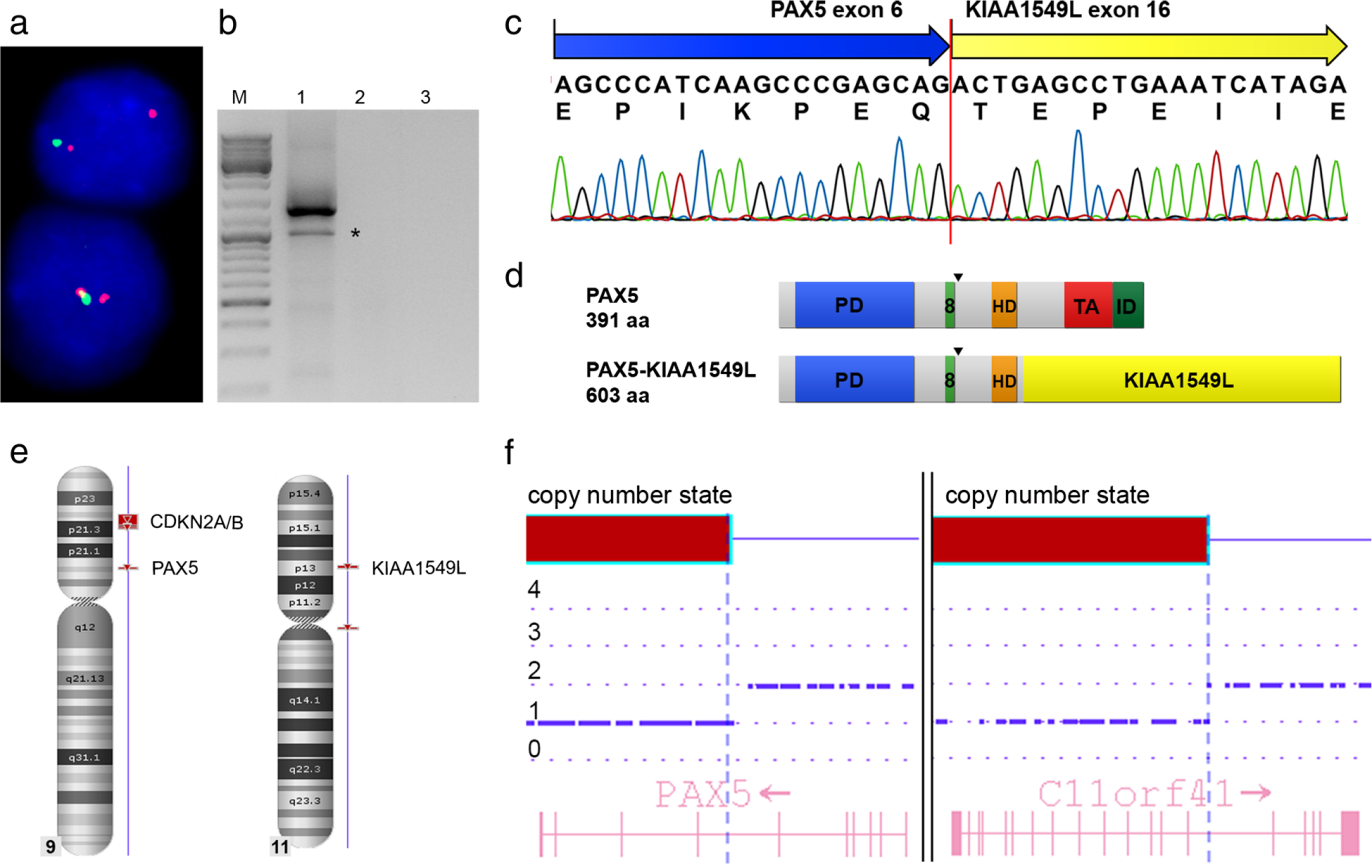
Cytogenetic and molecular analysis of *PAX5-KIAA1549L.* **a** Interphase FISH using *PAX5* exon-specific cosmid probes cos-hPAX5-1 (exons 2–5, red signals) and cos-hPAX5-3 (exons 9–10, green signals) showing loss of a *PAX5* 3’-end. **b** RT-PCR using primers specific for *PAX5* exon 5 and *KIAA1549L* exon 20 resulting in amplification of *PAX5-KIAA1549L* fusion transcripts. Sequencing of the second smaller band, indicated by an asterisk (*), revealed a splice variant of *PAX5-KIAA1549L*, lacking exon 16 of *KIAA1549L*. M, molecular weight marker DNA ladder-mix (Peqlab); lane 1, patient; lane 2, normal control; lane 3, water control. **c** Sequence analysis of the predominant RT-PCR product showing the presence of an in-frame fusion between *PAX5* exon 6 and *KIAA1549L* exon 16. **d** Schematic representation of the PAX5 wild-type and the PAX5-KIAA1549L proteins. PD, paired domain; 8, octapeptide; HD, partial homeodomain; TA, transactivation domain; ID, inhibitory domain; arrows indicate the nuclear localization signal of PAX5; aa, amino acids. **e** Chromosomes 9 and 11 with areas of genetic loss (red) detected by the CytoScan HD array and selected genes included in these areas. **f** Detailed view of *PAX5* on chromosome 9 (left panel) and *KIAA1549L* on chromosome 11 (right panel). The blue dashed lines indicate the breakpoints and the transitions from areas with normal and deleted (red bars) copy number states. The retained exons 1–6 of *PAX5* and exons 16–20 of *KIAA1549L* correspond to the fusion gene detected by RT-PCR

FISH analysis of the diagnostic bone marrow using *PAX5*-specific probes showed a deletion of the PAX5 3’-end in 72 % of the interphase cells, which is highly suggestive of the presence of a *PAX5* fusion gene (Fig. 1a). 3’-RACE-PCR and subsequent verification by RT-PCR identified *KIAA1549L* (*aliasC11orf41*)*,* located at 11p13, as novel *PAX5* fusion partner (Fig. 1b). Sequence analysis revealed an in-frame *PAX5-KIAA1549L* fusion transcript in which exon 6 of *PAX5* is fused to exon 16 of *KIAA1549L* (Fig. 1c). The predicted fusion protein retains the DNA-binding paired domain (PD), the octapeptide motif, the nuclear localization signal, and the homeodomain of PAX5, which are fused to the C-terminus of KIAA1549L (Fig. 1d).

KIAA1549L is a 1849 amino acids long protein with highly conserved regions whose functions have not been determined yet. As predicted by protein motif search, KIAA1549L harbors only a potential transmembrane domain encoded by exon 10, which, however, is not retained in the PAX5-KIAA1549L chimera. KIAA1549L is preferentially expressed in the human cerebral cortex and immunohistochemistry data suggest that KIAA1549L is localized in the nucleus as well as in cytoplasmatic vesicles [26, 27].

The opposite orientation of the partner genes – *PAX5* centromere-telomere and *KIAA1549L* telomere-centromere – excludes the formation of the *PAX5*-*KIAA1549L* fusion by a simple reciprocal translocation and their location and orientation suggested the presence of a dic(9;11) chromosome. Since the lack of metaphases precluded a detailed FISH analysis of the chromosomes involved in the rearrangement, we analyzed the genomic DNA using the CytoScan HD array, which – apart from the genome-wide detection of copy number alterations (CNAs) – permits to uncover unbalanced translocations. However, in contrast to *PAX5-ETV6* and *PAX5-C20orf112*, which are generated by the formation of dic(9;12) and dic(9;20) chromosomes, respectively [12, 28], in case of *PAX5-KIAA1549L* the CNAs did not corroborate the presence of a dic(9;11). Instead of losses of almost the entire p-arms of chromosomes 9 and 11, which would reflect the presence of a dic(9;11), we observed only minor deletions on the involved chromosomes (Fig. 1e). Consequently, similarly to other *PAX5* fusions [4, 8], also the *PAX5-KIAA1549L* fusion is most likely generated by a complex rearrangement.

A more detailed analysis of the CNAs of chromosomes 9 and 11 revealed a 324 kb heterozygous deletion on chromosome 9, which – amongst other genes – affected *PAX5* exons 7–10, and a 578 kb heterozygous deletion on chromosome 11, including *KIAA1549L* exons 1–15 (Fig. 1f). In line with the molecular analysis of the *PAX5-KIAA1549L* fusion gene (Fig. 1c), *PAX5* exons 1–6 and *KIAA1549L* exons 16–20, which are present in the fusion gene, were retained (Fig. 1f). These data indicate that only *PAX5-KIAA1549L* but no reciprocal transcripts are expressed, which supports the previous finding that in most *PAX5*-rearranged cases only *PAX5* fusion transcripts but no corresponding reciprocal ones are detectable [4, 8, 29].

**Fig. 2:**
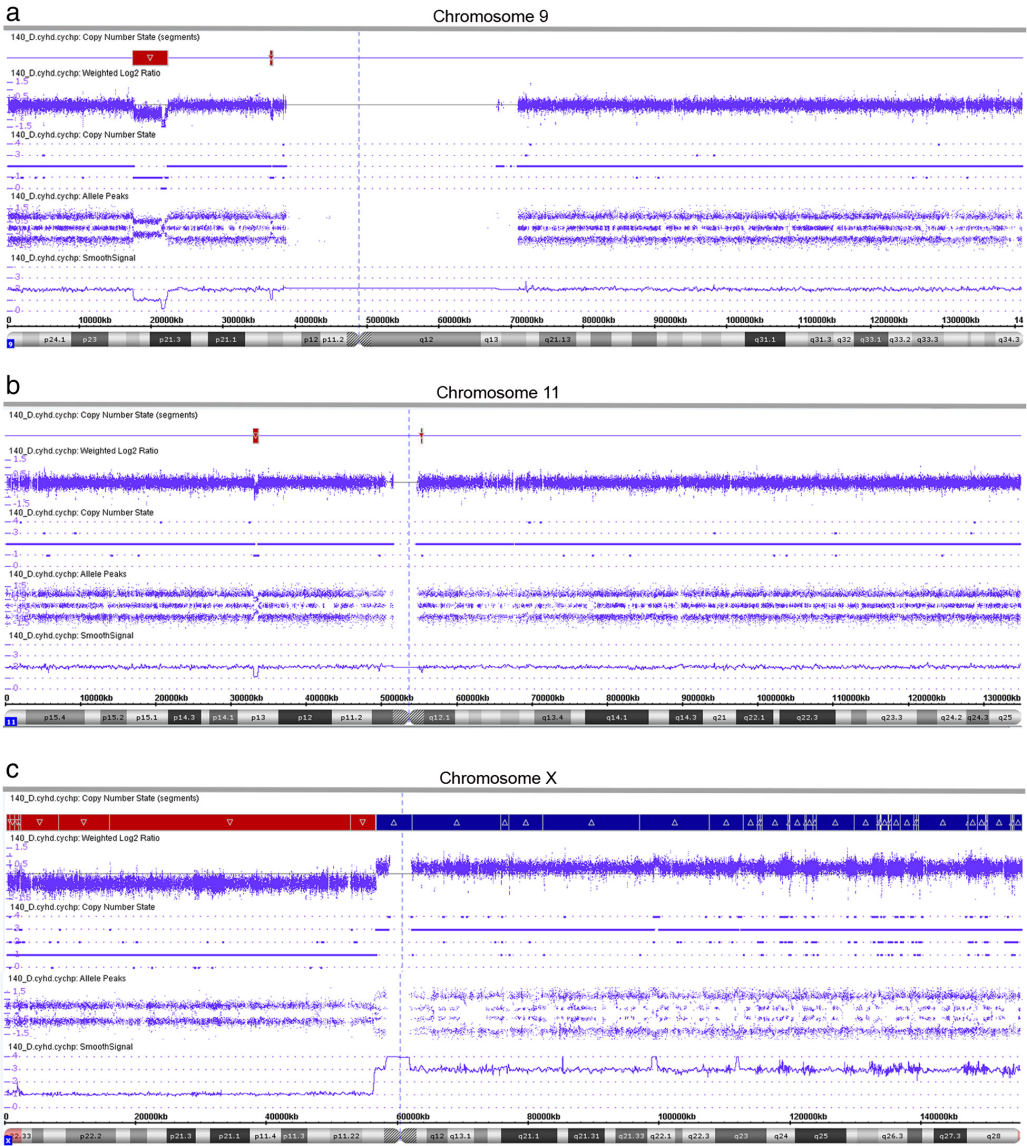
Detailed view of gains and losses of chromosomes 9, 11, and X. **a** Chromosome 9. The heterozygous 4760 kb deletion includes a homozygous deletion of *CDKN2A/B* indicated by the decrease of the smooth signal to zero. The smaller heterozygous loss affects, amongst other genes, the 3’-end of *PAX5*. **b** Chromosome 11. The heterozygous deletion at 11p13 includes the 5’-end of *KIAA1549L*. **c** Chromosome X. The changes of the log2 ratios along the chromosome indicate a trisomy of the entire q-arm and a small part of the p-arm as well as loss of most of the p-arm, indicating the presence of an idic(X)(p11)

With regard to other CNAs and in line with previous findings in *PAX5*-rearranged leukemia [8], we generally detected only a few minor gains and losses (Additional file 1). Chromosome 9 showed an additional heterozygous loss of a 4760 kb region at 9p21, within which we observed a smaller homozygous deletion affecting *CDKN2A/B* (Fig. 1e, Fig. 2a, Additional file 1). *CDKN2A/B* is commonly deleted in ALL [30] and its hetero- or homozygous loss is also frequently observed in dic(9;20) leukemia [31–33], which in some cases expresses the *PAX5-C20orf112* fusion gene [12, 32, 34, 35]. Furthermore, formation of the dic(9;12) resulting in the expression of the *PAX5-ETV6* fusion is invariably associated with a heterozygous deletion of *CDKN2A/B* as well [28, 35]. Therefore, loss of *CDKN2A/B* may represent a secondary genetic lesion that cooperates at least with a subset of *PAX5* fusions in the development of BCP-ALL.

Remarkably, chromosome X displayed a heterozygous loss of almost an entire p-arm and a gain of one copy of the entire q-arm, indicating the presence of an isodicentric idic(X)(p11) chromosome (Fig. 2c, Additional file 1). While idic(X)(q13) chromosomes are recurrently found in myeloid malignancies [36], an idic(X)(p11) is an extremely rare chromosome abnormality, which according to the Mitelman database [37] has only been described in a single case of Burkitt lymphoma [38].

Since *PAX5* fusions in ALL are rather rare [4, 5], their prognostic relevance *per se* and also the impact of secondary alterations including loss of *CDKN2A/B* remain to be determined. However, in this context it appears that *PAX5* deletions and mutations are not associated with clinical outcome [39] and that the prognostic value of *CDKNA/B* deletions is cytogenetic subtype and copy number alteration profile dependent [30, 40].

Involvement of *KIAA1549L* in another chromosome translocation has been previously reported in an adult case of acute myeloid leukemia with a t(11;21)(p14;q22) resulting in a *RUNX1-KIAA1549L* fusion [41]. In contrast to the *RUNX1-KIAA1549L* fusion, in which alternatively spliced *RUNX1* sequences are fused to exon 13 of *KIAA1549L, PAX5* exon 6 is fused to exon 16, indicating different genomic breakpoints in introns 12 and 15 of *KIAA1549L*, respectively. Furthermore, *KIAA1549*, a homolog of *KIAA1549L*, is involved in the recurrent *KIAA1549-BRAF* fusion gene found in pediatric and adult pilocytic astrocytoma [42].

In summary, we here describe a novel *PAX5* fusion partner and the second involvement of *KIAA1549L* in a leukemic fusion gene, indicating that it may play a so far underestimated role in the development of acute leukemia.

## Conclusion

In this study we identified *KIAA1549L* as novel *PAX5* fusion partner, which, on the one hand, increases the number of *PAX5* fusion partners found in BCP-ALL, and, on the other hand, shows that *KIAA1549L* is involved in at least two different fusion genes, emphasizing its potential role in the pathogenesis of acute leukemia.

## Consent

The patient was enrolled and treated with informed consent in the ALL-BFM 95 study (ClinicalTrials.gov Identifier: NCT00411541). This study was exclusively performed on material obtained for diagnostic purposes and neither any additional medical intervention nor patient recruitment was necessary.

## Additional file

Additional file 1:
**Summary of copy number alterations.**
