# Supplementary material for: PAX5-KIAA1549L: a novel fusion gene in a case of pediatric B-cell precursor acute lymphoblastic leukemia
Source: Mol Cytogenet. 2015 Jul 8;8:48. doi: 10.1186/s13039-015-0138-3 (PMC4495688; doi:10.1186/s13039-015-0138-3)
Supplement: Additional file 1: — Summary of copy number alterations. [file 13039_2015_138_MOESM1_ESM.pdf]

Additional file 1: Summary of copy number alterations detected by CytoScan HD Array

CN State	Type	Chr.	Cytoband Start	Cytoband End	Size (kb)	Marker Count	Gene Count	Genes	sno/mi RNAs	Microarray Nomenclature
3	Gain	2	q35	q35	63	60	2	PNKD, C2orf62		arr[hg19] 2q35(219,176,548-219,239,910)x3
1	Loss	3	p14.2	p14.2	512	946	1	FHIT		arr[hg19] 3p14.2(60,066,843-60,578,575)x1
3	Gain	4	q12	q12	78	88	1	PDGFRA		arr[hg19] 4q12(55,042,773-55,121,258)x3
1	Loss	9	p21.3	p21.3	320	273	2	CDKN2B-AS1, CDKN2B		arr[hg19] 9p21.3(22,003,246-22,323,201)x1
0	Loss	9	p21.3	p21.3	532	532	8	IFNE, MTAP, C9orf53, CDKN2A, CDKN2B-AS1, CDKN2B	MIR31HG, MIR31	arr[hg19] 9p21.3(21,471,028-22,003,123)x0
1	Loss	9	p22.2	p21.3	3908	4158	36	SH3GL2, ADAMTSL1, FAM154A, RRAGA, HAUS6, PLIN2, DENND4C, RPS6, ACER2, SLC24A2, MLLT3, FOCAD, PTPLAD2, IFNB1, IFNW1, IFNA21, IFNA4, IFNA7, IFNA10, IFNA16, IFNA17, IFNA14, IFNA22P, IFNA5, KLHL9, IFNA6, IFNA13, IFNA2, IFNA8, IFNA1	MIR3152, MIR4473, MIR4474, MIR31HG, MIR491, SCARNA8	arr[hg19] 9p22.2p21.3(17,562,988-21,470,997)x1
3	Gain	17	p13.1	p13.1	89	80	1	CCDC42		arr[hg19] 17p13.1(8,562,377-8,651,062)x3
3	Gain	7	q11.23	q11.23	140	224	4	GTF2I, NCF1, GTF2IRD2, STAG3L2		arr[hg19] 7q11.23(74,162,950-74,303,293)x3
1	Loss	9	p13.2	p13.2	324	408	5	MELK, PAX5	MIR4475, MIR4540, MIR4476	arr[hg19] 9p13.2(36,616,887-36,940,395)x1
1	Loss	11	p13	p13	587	502	6	TCP11L1, LINC00294, CSTF3, LOC338739, HIPK3, C11orf41		arr[hg19] 11p13(33,067,846-33,646,146)x1
1	Loss	X	p22.33	p11.21	56287	66840	382	not listed	not listed	arr[hg19] Xp22.33p11.21(168,546-56,455,292)x1
3	Gain	X	p11.21	p11.1	1906	1236	9	UBQLN2, LOC550643, UQCRBP1, SPIN3, SPIN2B, SPIN2A, FAAH2, ZXDB, ZXDA		arr[hg19] Xp11.21p11.1(56,457,791-58,364,114)x3
3	Gain	X	q11.1	q28	93310	121618	619	not listed	not listed	arr[hg19] Xq11.1q28(61,923,812-155,233,731)x3

Detailed overview of all copy number alterations (CNAs) detected by ChAS using the following settings: Genome segment filters were set to a marker count of 50 and a size of 50 kb for gains and losses. The ChAS Browser Annotations version NetAffx built 32.3 (UCSC genome assembly hg19) was used for analysis. CNAs have been checked for copy number variations (CNVs) as displayed by the ChAS software and compared to public databases including the Database for Genomic Variants (DGV) and the Database of Genomic Structural Variation (dbVar). All CNVs, which were described in normal control samples, were excluded.
